# Dataset for holiday rentals’ daily rate pricing in a cultural tourism destination

**DOI:** 10.1016/j.dib.2019.104697

**Published:** 2019-10-22

**Authors:** Miguel Ángel Solano Sánchez, Julia Margarita Núñez Tabales, José María Caridad y Ocerin, José António C. Santos, Margarida Custódio Santos

**Affiliations:** a Universidad de Córdoba, Facultad de Derecho y Ciencias Económicas y Empresariales, Spain; b Universidade Do Algarve, Research Centre for Tourism, Sustainability and Well-being (CinTurs), Portugal

**Keywords:** Holiday rental, Daily rate pricing, Sharing economy, Booking.com, Rental pricing

## Abstract

This data article describes a holiday rental dataset from a medium-size cultural city destination. Daily rate and variables related to location, size, amenities, rating, and seasonality are highlighted as the main features. The data was extracted from Booking.com, legal registration of the accommodation (RTA) and Google Maps, among other sources. This dataset contains data from 665 holiday rentals offered as entire flat (rent per room was discarded), with a total of 1623 cases and 28 variables considered. Regarding data extraction, RTA is ordered by registration number, which is taken and, through a Google search with the following structure: “apartment registration no. + Booking + Seville”, the holiday rental profile in Booking.com is found. Then, it is verified that both the address of the accommodation and the registration number match in RTA and Booking.com, proceeding with data extraction to a Microsoft Excel's file. Google Maps is used to determine the minutes spent walking from the accommodation to the spot of maximum tourist interest of the city. A price index based on the average price per square meter of real estate per district is also incorporated to the dataset, as well as a visual appeal rating made by the authors of every holiday rental based on its Booking.com photos profile. Only cases with complete data were considered. A statistics summary of all variables of the data collected is presented. This dataset can be used to develop an estimation model of daily prices of stay in holiday rentals through predetermined variables. Econometrics methodologies applied to this dataset can also allow testing which variables included affecting the composition of holiday rentals' daily rates and which not, as well as determining their respective influence on daily rates.

**Table:** Specifications Table

Subject	Tourism, Leisure and Hospitality Management
Specific subject area	Holiday rentals' daily rate pricing
Type of data	Table
How data were acquired	Extracted from Booking.com through Google searches
Data format	Raw
Parameters for data collection	All holiday rentals legally registered in Seville designated in Spanish as “viviendas con fines turísticos” (VFT) (i.e. homes for tourism purposes), offering the full modality (i.e. the entire flat) were taken as total research population, based on “Registro de Turismo de Andalucía” (RTA) (i.e. the Andalusian Tourism Registry). Only cases with complete data were considered. The final sample included the same lodgings with different offers regarding the number of beds.
Description of data collection	The VFT registration in RTA is ordered by registration number. The registration number is taken and a Google search is made following this structure: “[VFT registration code] + Booking + Seville”. It is verified that both the address of the VFT and the registration number match in RTA and Booking.com. All the data of the variables considered of the VFTs are extracted and the price is taken in different time periods (see Table 2).
Data source location	The city of Seville, Andalusia, Spain
Data accessibility	The complete raw dataset is provided as supplementary Excel table within the article

**Table:** 

**Value of the Data** • An estimation model of daily prices of stay in holiday rentals through predetermined variables can be developed through this dataset; • The data can benefit both owners and users of holiday rentals, helping these individuals to determine if their price of stay is what would commonly be offered in the market under normal circumstances; • This dataset allows testing which variables included affecting the composition of holiday rentals' daily rates and which not through econometrics methodologies as hedonic regressions; • This dataset allows to determine the individual influence of the relevant variables on holiday rentals' daily rates through econometrics methodologies as hedonic regressions; • Descriptive analytics can be employed to further understand patterns, trends, and anomalies in data.

## 1 Data

The dataset contains raw data from 665 Sevillian holiday rentals (Table 1) offered as entire flat extracted mainly through Booking.com searches, among other sources (Table 2). The Microsoft Excel worksheet provided as supplementary data for this article (see Appendix A) includes the complete dataset of 1623 cases and 28 variables (Table 2) considered. Summary statistics of the dataset's numerical and integer (Table 3) and categorical (Table 4) variables are also presented. Finally, Table 5 shows how DINDEX variable is constructed.

**Table 1:** Population, sample, and cases in the dataset.

Total number of VFTs registered in Seville [2] (October 2018)	3750
VFT in full modality rented	3467
Sample of VFT with complete data	665
Total number of cases incorporated to the dataset	1623

**Table 2:** Variables description.

Variable	Type	Description	Source
REG	Categorical	RTA registration code no.	[2,3]
ADD	Categorical	VFT Address	[2,3]
PRICE	Numerical	Daily rate (in €) for a two-day stay (average stay in Seville [4])	[3]
MIN	Integer	Minutes to walk from VFT to spot of maximum tourist interest (i.e. Plaza del Triunfo, as is located between the Cathedral of Seville and the Real Alcázar, the two most visited monuments in Seville [4]). Variables regarding distance to the centre/place of interest are in the same line of [5].	[6]
DISTRICT	Categorical	District where the VFT is located	[3]
DINDEX	Numerical	District index was constructed from the average price per m^2^ of housing in Seville according to the district where the accommodations are located. The district with the highest price took value 1, and the rest of the districts acquired a proportional value according to their prices (see Table 5).	[7]
BEDS	Integer	Number of beds	[3]
M2	Integer	Square meters	[3]
TV	Categorical	TV (No = 0; Yes = 1)	[3]
WASHM	Categorical	Washing machine (No = 0; Yes = 1)	[3]
BALCONY	Categorical	Balcony (No = 0; Yes = 1)	[3]
TERRACE	Categorical	Terrace (No = 0; Yes = 1)	[3]
CRTYD	Categorical	Courtyard (No = 0; Yes = 1)	[3]
VIEWS	Categorical	Views (No = 0; Yes = 1)	[3]
SND	Categorical	Soundproofing (No = 0; Yes = 1)	[3]
PARK	Categorical	Parking (No = 0; Yes = 1)	[3]
PETS	Categorical	Pets allowed (No = 0; Yes = 1)	[3]
POOL	Categorical	Pool (No = 0; Yes = 1)	[3]
BATH	Categorical	Bathtub (No = 0; Yes = 1)	[3]
RAT	Numerical	Rating of previous users (from 0 to 10)	[3]
PICS	Integer	Number of photos	[3]
VSAP	Numerical	Visual appeal according to photos (from 0 to 10)	Authors
HSWD	Categorical	High season, weekday (price from 27/05/2019 to 29/05/2019) (No = 0; Yes = 1)	[3]
HSWE	Categorical	High season, weekend (price from 31/05/2019 to 02/06/2019) (No = 0; Yes = 1)	[3]
LSWD	Categorical	Low season, weekday (price from 14/01/2019 to 16/01/2019) (No = 0; Yes = 1)	[3]
LSWE	Categorical	Low season, weekend (price from 18/01/2019 to 20/01/2019) (No = 0; Yes = 1)	[3]
SE1	Categorical	Special Event 1 (Holy Week in Seville) (price from 18/04/2019 to 20/04/2019) (No = 0; Yes = 1)	[3]
SE2	Categorical	Special Event 2 (April Fair in Seville) (price from 10/05/2019 to 12/05/2019) (No = 0; Yes = 1). In the same line of [8].	[3]

**Table 3:** Dataset summary statistics of numerical and integer variables.

Variable	Mean	SD	Min. Value	Max. Value
PRICE	162,09	105,54	42,00	1164,90
MIN	14,71	8,53	1	93
DINDEX	0,96	0,09	0,41	1,00
BEDS	3,94	1,90	1	15
M2	75,80	40,81	9	400
RAT	8,87	0,68	0	10
PICS	32,80	11,22	3	56
VSAP	8,40	0,83	0	10

**Table 4:** Dataset summary statistics of categorical variables.

Variable	Counts	% over 1623 cases
DISTRICT (Casco Antiguo)	1301	80,16%
DISTRICT (Triana)	207	12,75%
DISTRICT (Nervión)	55	0,03%
DISTRICT (Macarena)	22	0,01%
DISTRICT (Los Remedios)	14	0,01%
DISTRICT (San Pablo/Santa Justa)	11	0,01%
DISTRICT (Sur)	6	0,00%
DISTRICT (Este/Alcosa/Torreblanca)	3	0,00%
DISTRICT (Bellavista/La Palmera)	2	0,00%
DISTRICT (Cerro Amate)	2	0,00%
DISTRICT (Norte)	0	0,00%
TV	1613	99,38%
WASHM	1555	95,81%
BALCONY	719	44,30%
TERRACE	580	35,74%
CRTYD	549	33,83%
VIEWS	868	53,48%
SND	339	20,89%
PARK	653	40,23%
PETS	186	11,46%
POOL	41	2,53%
BATH	554	34,13%
HSWD	565	34,81%
HSWE	218	13,43%
LSWD	465	28,65%
LSWE	167	10,29%
SE1	129	7,95%
SE2	79	4,87%

**Table 5:** DINDEX elaboration.

District	€/m^2^	DINDEX
Casco Antiguo (Old Town)	2398	1
Los Remedios	2196	0,916
Nervión	2137	0,891
Triana	1932	0,806
Sur	1825	0,761
San Pablo/Santa Justa	1629	0,679
Bellavista/La Palmera	1578	0,658
Macarena	1301	0,543
Este/Alcosa/Torreblanca	1226	0,511
Norte	1041	0,434
Cerro Amate	974	0,406

Source: authors based on [7].

Table 1 presents the population, sample and the total number of cases included in the dataset. In the Andalusian legislation regulating holiday rentals in Seville [1], these accommodations are designated as “Viviendas con Fines Turísticos” (VFT) (i.e. homes for tourism purposes). A VFT can be rented in full (i.e. the entire flat) or in part (i.e. a spare room). All legally registered VFT [2] were considered, excluding the spare room rented modality. The number of cases includes same VFT offered at different prices regarding the number of beds. Table 2 shows all variables considered in the dataset, its type, description, and source.

## 2 Experimental design, materials, and methods

First, based on RTA register [2], VFT code and its address are copied and displayed in ascending order by VFT code through a Microsoft Excel worksheet (the dataset presented in the article). Second, one by one, a google search is started following the structure: “[VFT registration code] + Booking + Seville”. Third, a click is made on the Booking.com VFT profile and is checked that both the VFT code and its address match in RTA and Booking.com. Then, all the variables considered available on Booking.com (see Table 2) are extracted and copied into the Microsoft Excel worksheet file. VFTs with incomplete data are discarded.

The daily rate is copied in all the different time periods considered (HSWD, HSWE, LSWD, LSWE, SE1, and SE2; see Table 2) and later weighted (see Table 4) in order to get a sole PRICE variable. Once this process is finished, through Google Maps searches [6], MIN (Table 2) is obtained one by one and copied into the aforementioned Microsoft Excel file. DINDEX variable is filled following the criteria described in Table 2 with the data obtained in Table 5. Finally, all the VFT photos available in their own Booking.com profile are observed, and each VFT is rated by the authors regarding its visual appeal (VSAP, Table 2).

To conclude, a statistical summary of the numerical, integer (Table 3) and categorical (Table 4) variables included in the dataset are presented.
